# Principles Involved in the Law of Cure

**Published:** 1893-11

**Authors:** W. E. Ledyard

**Affiliations:** San Francisco, Cal.


### PRINCIPLES INVOLVED IN THE LAW OF CURE.

Dr. W. E. Ledyard, San Francisco, Cal.

The writer will in this article attempt to show *what* these principles are, and *how* they may be *helped* or *hindered*.

1. By the *air* we breathe;
2. By the *clothing* we wear;
3. By the *houses* we live in;
4. By the *food* we eat;
5. By our *mode of life;*
6. By our *occupation;*
7. By our *mode of travel;*
8. By the *medicine* we take, and
9. By the *thousand-and-one nameless little things that go to make up life.*

1. It is a foregone conclusion that *the air we breathe* must be fresh and pure, if we would retain our health.

In the home; traveling daily (as so many do nowadays in going to and from their places of business) by land or water; in the church or public hall—many prefer to have doors and windows tightly closed.

These people consider themselves fortunate in securing a seat in the over-crowded and frequently over-heated saloon of a ferry-boat, where the oxygen of the atmosphere is removed by the first inspiration, and every succeeding breath drawn is vitiated more and more with the poison-laden carbonic acid gas and other effete matters which are constantly being thrown off by many pairs of lungs, and often quite perceptibly by the skin.

They honestly believe this to be the best way to keep what little health they have.

The reason why they prefer the unhealthy atmosphere to the fresh air is easily found in the dread of "taking cold," and thus falling an easy prey to the grippe, pneumonia, "*et hoc genus omne.*"

Perhaps you will say: "Is not this a reasonable fear, and are they not in danger of 'taking' one or more of the above diseases, if they are sufficiently indiscreet to expose themselves?"

To which we readily answer, "Yes!"

However, we shall now attempt to show that the danger originated in themselves. By always carefully avoiding every breath of fresh air, wrapping up and coddling themselves, they have become *so susceptible to cold* that the slightest exposure is almost sure to make them very sick, and their *want of recuperative power* is all-sufficient to keep them sick.

Compare, if you please, these people with those who are not afraid of fresh air, and who always avoid the close and poison-laden atmosphere, wherever found.

The latter seldom get sick, and even when they do, their recuperative power is such that they are soon well.

There is, then, a decided difference in these two classes. To explain the reason for this difference, we shall say something about the *recuperative power* to which allusion has been made. We shall refer to this under the name of "vital force."

Every one when created is endued with a certain amount of vital force. In a state of perfect health, all the parts and organs of the body act with such harmony that they would appear to have no existence, so far, at least, as the sensations are concerned.

However, the moment the fell disease asserts himself this harmony is disturbed, the equilibrium is lost, and the departure from health is inaugurated by painful sensations.

The cause of this disturbance, or disease—*i. e.*, want of ease—is the action of the above-mentioned vital force in its efforts to re-establish the lost equilibrium and thus restore health.

To regain health in the quickest manner possible, it is necessary to assist and not to resist the all-important vital force.

If such be the case, then anything and everything that interferes in any way with the action of the vital force should, as far as possible, be avoided.

Any close atmosphere, to a great extent, prevents the action of the vital force—*i. e.*, prevents nature's efforts to restore the lost balance—in other words, to bring about a return to health.

Possibly we can make this point plainer by illustration :

The tree, standing alone in a situation where it is exposed to storms of wind and rain, *sends its roots a little deeper and takes a firmer hold with each fresh blast that sweeps over it*, and thus, by opposing strength to strength, becomes itself all the stronger and firmer.

If it were possible to transplant a full-grown tree from the midst of a forest, where it is surrounded on all sides by other trees, to this exposed site, in all probability the first severe storm would be more than sufficient to lay it in the dust.

Similarly, when one takes an outside seat on a ferry-boat, day after day and week after week, he becomes like the above tree, and the storms of the vital force have full play.

At first there is a slight chilliness, perhaps a cough, possibly a sneeze.

This is the action of the vital force as it resists the threatened evil—the signal of distress calling loudly for help—the danger-flag pronouncing something wrong in the ship—the alarm-bell ringing out its notes of warning "with no uncertain sound."

How shall we best respond to this urgent call and assist the vital force?

Shall we go within, so that the cough may stop and the chilliness disappear?

We have done so. The cough subsides and also the chilliness, but *the cause is still there*, for, as soon as we re-enter the cool air, the *cough returns and becomes, if anything, more persistent.*

By going inside we have torn down the danger-flag, we have muffled the alarm-bell.

As we re-enter the open air, up goes the flag in the shape of a cough—off goes the muffle as a sneeze.

There is only one opinion possible with regard to the sailor who tears down the danger-flag, and then insists that there is no danger because no flag.

2. *The clothing we wear* may have much to do in helping or hindering the proper action of the *vital force.*

This is the life-giving power by which we, under God, "live, move, and have our being," and it must not be thought of simply in connection with disease.

As a notable example of improper clothing, take *the corset.*

If you wish to prove that the corset is injurious to the health, ask the person who wears it constantly why she does so. She will tell you, without a moment's hesitation, that it is because it is *such a support to the back.*

Now you have the key to the whole situation. The opposite sex, who are not in the habit of wearing the article of apparel which is now calling forth our disapproval, do not complain of want of support to the back.

The spine and muscles of the back constitute the natural supports of the back. When, however, the constricting and paralyzing corset has been worn for a length of time, and has, like the wily serpent, almost squeezed the life out of these parts, the muscles, as a matter of course, lose all power of action, this power having been relegated to the poor, inefficient corset.

Consequently, when the latter is laid aside, even for a short time, the muscles fail to respond, because, through long inaction, they have lost the power to act, and are, to all intents and purposes, as if they did not exist, like the fishes that swim in the dark waters of some subterranean cave, that, having no use for eyes, are born without these, to them, useless organs.

In this connection, it is also well to remember what part clothing is designed to fulfill in our economy.

Its use, then, is principally as a *covering* for the body, to protect it from the injurious effects of heat and cold, to be fashioned in such a manner that each part of the body has perfect freedom of action.

*Tightness of clothing* in any part interferes with the action of the vital force, preventing the free circulation of the life-giving stream, and the proper supply of sensation to the parts.

And this is the great objection to every *mechanical apparatus* for the support of spine, limbs, or other parts.

The latter absolutely deprives the vital force of all power to act, and thus *prevents what it is intended to accomplish.*

We have known a physician to give what he considered the indicated remedy for pleuritic pain in the chest. But, in order "to make assurance doubly sure," he took the precaution to help out his own meagre supply of faith in the remedy by applying a bandage tightly around the chest.

To us, this is a case of "falling to the ground between two stools"—the medicine and the bandage—the latter, like the corset, doing the work in a poor, ineffective way, while the former, like Othello, is left "without an occupation."

Here we may well pause to inquire how often *we*, as well as our patients, *prevent the action of the indicated remedy* by interposing some poor, innocent-looking make-shift, *which certainly removes the distressing symptoms, but does so in a wrong way*—*i. e.*, without first removing the cause, which would be the case if the *indicated* remedy were not interfered with.

Pessaries, supports of all kinds, local applications, strong scents used about the person, tooth powders, etc., have the same tendency to suppress diseases.

3. *The houses we live in.*

"It goes without saying" that these should be favorably situated, clean, sunny, roomy, and well-ventilated.

The rays of light and sunshine should never be carefully excluded from the "best room," nor should heavily-curtained windows be allowed.

Never close doors and windows at the expense of fresh air simply to "keep out the flies."

Accomplish this object by supplying screens to doors and windows.

Carpets should be taken up and thoroughly cleaned at least twice a year.

Beds should be very comfortable—not so luxurious as to be enervating. Feather-beds and too many bed-covers have this effect.

Bed-clothing should be well aired for several hours every day.

If possible, do not sleep in the clothes you wear in the daytime.

Allow no decaying vegetable matter or any harbor for moths to remain upon the premises.

Have your houses beautiful with paintings and decorations of all kind, especially let them be beautiful with holiness—places where the milk of human kindness flows freely—perfect little paradises to which tired husbands may gladly turn their weary steps.

4. *The food we eat.*

This is by no means the smallest factor in the solution of the principles we are now studying.

Nothing is more common than to hear of people with whom certain kinds of food appear to disagree. Indeed, so much is this the case that it has called forth the expression: "What's one man's food is another man's poison."

In the face of this very positive assertion of the mighty authority designated "public opinion," we venture to state that *all* food was given to us for our benefit by an all-wise Creator.

When food disagrees, one of two things is usually at fault: *the eater or the thing eaten.*

If the former, it will disagree with that individual only; if the latter, every one who partakes will be affected.

Nine times out of ten the thing eaten receives the blame, and presumably the only way out of the difficulty is to avoid that particular article of diet.

If the trouble is in the food there is something wrong with *it*—with that particular article—surely not with every article bearing the same name. Then procure a good specimen of the same, and *if that disagrees, the trouble is in the eater, not in the thing eaten.*

Again, a person sits down to a hearty meal when highly *excited,* much *exhausted,* or very *angry.*

Something on the table, as he supposes, disagrees with him.

The next time that particular article, which, in his opinion, has obtained the unenviable reputation of having kicked up such a rumpus in the inner-man, appears on the festive board, it is forthwith "boycotted," and studiously avoided, as one of the bug-bears of life.

Of course we can see that in this instance at least *the eater deserves all the blame.*

Excitement, exhaustion, or anger is a poor preparation for digesting one's food, and will undoubtedly interfere with or even stop digestion at any time, and has caused serious, sometimes fatal, effects.

The mother, who, before recovering from a fit of anger or other emotional disturbance puts the infant to the breast, might as well give the child poison, for it will surely be thrown into convulsions or some equally serious effect will be produced.

The stomach is, as it were, left to itself, unaided by the blood and nerve supplies, which are elsewhere, paying strict attention to the object which has inspired the anger, etc.

The food enters the stomach, which is thus completely unprepared for its reception.

In the attempt to recover from this surprise, something is bound to give way, and *presto!* a fit of indigestion or some other untoward result.

All this might be avoided by patiently waiting for twenty minutes or half an hour.

There are many persons in whom certain kinds of food are invariably followed by certain painful effects. On this account many *physicians advise* such persons *to avoid all such articles of diet.*

Let us investigate and endeavor to discover if such advice be good or bad.

The articles alluded to are avoided, and the patient is comparatively free from indigestion, but this is so *only so long as he abstains from the food which had before disagreed with him.*

Such a doubtful expedient he might and often does resort to without the physician's advice.

As the great father of Homœopathy says in the opening words of his never-to-be-forgotten *Organon*, "The first and sole duty of the physician is to restore health to the sick"—not to avoid everything that makes sick, which is merely "begging the question," and altogether avoiding the main issue.

If the eater and not the thing eaten be at fault, then it is the physician's duty to rectify the former by the indicated remedy.

The trouble is usually in the *constitution* of the patient, or it may be in his mode of living, such as a sedentary occupation, excessive mental exertion, anxiety, the abuse of alcoholics or tobacco, excessive indulgence in anything, etc., etc.

5. *Our mode of life.*

We may have acquired very bad habits: eating or drinking hurriedly, partaking of unwholesome food, keeping late hours, lying in bed late, taking too little exercise, etc., etc.

6. *Our occupation* may be sedentary, thus tending to produce indigestion and other complaints, as in the seamstress and in the student.

It may be attended by *heavy lifting*, giving rise to sprains and strains. Excessive mental exertion, as in the great financier; anxiety, as in the case of the mother with a sick child; colic of the painter or plumber, etc.

In all of the above cases the occupation is hindering the action of the vital force.

*Healthy occupations* are such as those of the carpenter, farmer, or gardener, which give one plenty of exercise in the open air.

7. *Our mode of travel* we have briefly alluded to.

8. *The medicine we take.*

It is a "popular fallacy" to suppose that medicine, to cure, must be taken in its crude form, in massive doses, and that it must be possessed of color, a decidedly disagreeable taste, and a smell anything but pleasing to the olfactory nerves.

A drug, when taken into the system as above, must, in the very nature of things, produce its *peculiar* poisonous effect upon the system.

To act as a curative, it must be given in a *potentized* form and to a patient suffering from symptoms *similar* to those which the drug is capable of producing.

Nor is a *local application* of a crude drug in any manner curative but the reverse.

To have a right conception of disease, we must always remember that it *originates within the system*, and that the *eruption, ulcer*, or *discharge*, etc., is but the *external manifestation*, the sign, the evidence, if you please, of the internal disease.

This is the way in which our all-wise Maker and Preserver has ordained that we shall be able to recognize and cure diseases.

The ulcer, or other appearance on the skin, combined with all other symptoms, being *the only true guide in the selection of the remedy*, it follows, as a matter of course, that this symptom should be "left severely alone," because a *local* medicinal application, *by removing or changing the appearance or character of this our sole guide, will mar or obliterate the disease picture, and thus render it impossible to make a correct choice of the curative remedy.*

Moreover, the effort of the vital force itself has caused the external manifestation, that the internal disease may be relieved thereby. Therefore, it invariably happens that the general health of the patient is better when the ulcer, eruption, or discharge is fully established.

The external application, by stopping this discharge, for instance, *causes a suppression of the disease*, and the patient again becomes worse until the discharge is re-established.

9. *The thousand-and-one little things.*

Of these we shall only be able to mention two or three.

*Putting pledgets of cotton in the ears* is especially reprehensible, as it is the means of inviting earache and neuralgia by increasing the susceptibility to cold.

It also, in time, injures the sense of hearing, to say nothing of the danger arising from a foreign body impacted in the ear.

We have removed cotton from the interior of the ear, after it had been lying there for weeks, when the skunk would have been as attar of roses to the stench from the pus emanating therefrom—and the danger from meningitis or septicæmia was by no means remote.

For those who work in noisy places, as in the manufacture of boilers, we would strongly recommend a small pledget of cotton to be *loosely* inserted in the ear, on entering the factory, to be removed at once, after leaving the same.

This will prevent the deafness which affects all who are employed in such works.

The deafness arises in this way: The constant vibrations of sound beat against the sensitive tympanic membrane. In the ordinary healthy ear, this might rupture the membrane, but that the vital process, to prevent the possibility of such an occurrence, gradually thickens the membrane, which, of course, causes an ever-increasing deafness.

The cotton prevents the vibrations reaching the drum of the ear, and thus it is preserved in a healthy condition.

Removing ear-wax by means of a hair-pin, a bit of stick, tooth-pick, match end, etc., may cause irreparable injury to the sense of hearing through the same membrane.

Using pins which have been picked up in the street, and which, for all you know to the contrary, may have recently done duty in fastening the bandage over some festering ulcer, putting in the mouth coins which may have lately been handled by one suffering from a loathsome disease, drinking from the cup which may be used by anybody on train or ferry-boat or at drinking fountain on the street, using other people's hair-brushes or towels, are a few of the ways in which danger may insidiously arise.

We might incidentally allude to chewing gum, which passes from mouth to mouth around the school, and to the dangerous custom of indiscriminate kissing.

Possibly stone-cutters and workers in metal could, to a great extent, prevent the inhalation of particles by wearing a fine gauze veil or some closely-woven material which would freely admit the passage of air while it prevented the admission of the offending particle.

Our motto for all sick people is the same as that printed in large letters on some fragile piece of exquisite workmanship to preserve it from ruthless interference. The motto referred to is "Hands Off!" and we would have all patients who expect to be cured "*cito tuto et jucundo*" thus labeled. The remedy should be allowed to act unobstructed by any impediment of food, dress, mode of life, or aught else; then we may with Moses: "Stand still and see the salvation of the Lord."

